# Supplementary material for: Evidence from UK Research Ethics Committee members on what makes a good research ethics review, and what can be improved
Source: PLoS One. 2023 Jul 3;18(7):e0288083. doi: 10.1371/journal.pone.0288083 (PMC10317218; doi:10.1371/journal.pone.0288083)
Supplement: S1 Data — (ZIP) [file pone.0288083.s001.zip › Supplementary Data/Question 1/Ethics Review Form.docx]

Files\\Qu1 - § 10 references coded [ 13.33% Coverage]

Reference 1 - 1.33% Coverage

LRF on HARP

Reference 2 - 1.33% Coverage

In some RECs everyone has to put their comments on HARP LRF

Reference 3 - 1.33% Coverage

Look at the LRF sections or the study holistically?

Reference 4 - 1.33% Coverage

LRF most useful when not the lead/2nd reviewer to pick out main ethical issues

Reference 5 - 1.33% Coverage

Use the LRF form for ethical domains

Reference 6 - 1.33% Coverage

LRFs save time on discussions

Reference 7 - 1.33% Coverage

HARP LRF ok - option for a bottom box? Remove duplication e.g. insurance.

Reference 8 - 1.33% Coverage

LRF very useful for all members - gives a weighted voice.

Reference 9 - 1.33% Coverage

LRF - Pre Review on HARP - questions are posed on HARP before the meeting.

Reference 10 - 1.33% Coverage

Use the HARP LRF in advance of the meeting for reviews.
