# Supplementary material for: Evidence from UK Research Ethics Committee members on what makes a good research ethics review, and what can be improved
Source: PLoS One. 2023 Jul 3;18(7):e0288083. doi: 10.1371/journal.pone.0288083 (PMC10317218; doi:10.1371/journal.pone.0288083)
Supplement: S1 Data — (ZIP) [file pone.0288083.s001.zip › Supplementary Data/Question 1/RiskBenefit.docx]

Files\\Qu1 - § 4 references coded [ 5.33% Coverage]

Reference 1 - 1.33% Coverage

Deliberative decision-making process works well. John Frenham/Clive Collett reviewed ShEd outputs and found that ethical decisions were made not on moral grounds but more on group opinion and safeguarding. Studies which have already received NIHR, MRC review etc already carry an element of being low in ethical risk. Also mark Sheehan has written a paper showing that REC decision making is similar to Jury decision making in that it is deliberative.

Reference 2 - 1.33% Coverage

Do we look at ethics or safeguards?

Reference 3 - 1.33% Coverage

Risks and benefits? What are they? How are they outlined in the PIS?

Reference 4 - 1.33% Coverage

How is the patient affected?
