# Supplementary material for: Evidence from UK Research Ethics Committee members on what makes a good research ethics review, and what can be improved
Source: PLoS One. 2023 Jul 3;18(7):e0288083. doi: 10.1371/journal.pone.0288083 (PMC10317218; doi:10.1371/journal.pone.0288083)
Supplement: S1 Data — (ZIP) [file pone.0288083.s001.zip › Supplementary Data/Question 1/Proportionate Approach.docx]

Files\\Qu1 - § 3 references coded [ 3.93% Coverage]

Reference 1 - 1.33% Coverage

Be proportionate. Take a proportionate approach.

Reference 2 - 1.33% Coverage

May not come to a decisions early - it can take some time.

Reference 3 - 1.26% Coverage

Guidelines to use on specific documents
