# Supplementary material for: Evidence from UK Research Ethics Committee members on what makes a good research ethics review, and what can be improved
Source: PLoS One. 2023 Jul 3;18(7):e0288083. doi: 10.1371/journal.pone.0288083 (PMC10317218; doi:10.1371/journal.pone.0288083)
Supplement: S1 Data — (ZIP) [file pone.0288083.s001.zip › Supplementary Data/Question 1/Consistency.docx]

Files\\Qu1 - § 5 references coded [ 6.67% Coverage]

Reference 1 - 1.33% Coverage

Can be a lack of consistency across RECs – both outcomes and experience at the meeting.

Reference 2 - 1.33% Coverage

Approach as a new member has evolved with experience.

Reference 3 - 1.33% Coverage

FOCUS is more important than EFFICIENCY

Reference 4 - 1.33% Coverage

Getting to opinion depends on the REC membership.

Reference 5 - 1.33% Coverage

Guidance is needed on specialist areas like statistics.
