# Supplementary material for: Evidence from UK Research Ethics Committee members on what makes a good research ethics review, and what can be improved
Source: PLoS One. 2023 Jul 3;18(7):e0288083. doi: 10.1371/journal.pone.0288083 (PMC10317218; doi:10.1371/journal.pone.0288083)
Supplement: S1 Data — (ZIP) [file pone.0288083.s001.zip › Supplementary Data/Question 1/Focus on Research Design.docx]

Files\\Qu1 - § 6 references coded [ 7.83% Coverage]

Reference 1 - 1.33% Coverage

Well designed? Will it produce what they want it to produce?

Reference 2 - 1.33% Coverage

Well designed?

Reference 3 - 1.33% Coverage

Is there a simple starting point?

Reference 4 - 1.32% Coverage

The table agreed that the role of the REC was to concentrate on the ethical issues and not the methodology of the study. However, if the methodology was poor the study could be unethical. There are ways of asking about the methodology e.g. have you had a statistician review the methodology and is there an independent review.

Reference 5 - 1.28% Coverage

Put the research question into context - is it new or similar?

Reference 6 - 1.22% Coverage

do we consider the science
