# Supplementary material for: Evidence from UK Research Ethics Committee members on what makes a good research ethics review, and what can be improved
Source: PLoS One. 2023 Jul 3;18(7):e0288083. doi: 10.1371/journal.pone.0288083 (PMC10317218; doi:10.1371/journal.pone.0288083)
Supplement: S1 Data — (ZIP) [file pone.0288083.s001.zip › Supplementary Data/Question 1/Focus on PIS.docx]

Files\\Qu1 - § 7 references coded [ 9.20% Coverage]

Reference 1 - 1.33% Coverage

Some people start with the PIS (as if they were a participant), rather than the protocol.

Reference 2 - 1.33% Coverage

Treat each study as a patient. Read the PIS first and ICF. Then read the IRAS form and cross reference.

Reference 3 - 1.33% Coverage

Risks and benefits? What are they? How are they outlined in the PIS?

Reference 4 - 1.33% Coverage

Understand the study PIS?

Reference 5 - 1.32% Coverage

Is the PIS understandable to the average reader. For a 12 page PIS there are ways to make sure participants understand the requirements of the study by making sure the first page includes all the relevant information.

Reference 6 - 1.31% Coverage

The table agreed that it was really important for clinical trials for the REC to compare the PIS to the protocol as the MHRA do not do this.

Reference 7 - 1.24% Coverage

check the PIS, protocol and IRAS
