# Supplementary material for: Evidence from UK Research Ethics Committee members on what makes a good research ethics review, and what can be improved
Source: PLoS One. 2023 Jul 3;18(7):e0288083. doi: 10.1371/journal.pone.0288083 (PMC10317218; doi:10.1371/journal.pone.0288083)
Supplement: S1 Data — (ZIP) [file pone.0288083.s001.zip › Supplementary Data/Question 1/PPI.docx]

Files\\Qu1 - § 1 reference coded [ 1.29% Coverage]

Reference 1 - 1.29% Coverage

perspectives of the stakeholders/researchers/participants
