# Supplementary material for: Evidence from UK Research Ethics Committee members on what makes a good research ethics review, and what can be improved
Source: PLoS One. 2023 Jul 3;18(7):e0288083. doi: 10.1371/journal.pone.0288083 (PMC10317218; doi:10.1371/journal.pone.0288083)
Supplement: S1 Data — (ZIP) [file pone.0288083.s001.zip › Supplementary Data/Question 1/Zoom vs F2F.docx]

Files\\Qu1 - § 5 references coded [ 6.67% Coverage]

Reference 1 - 1.33% Coverage

MDT aspects of REC most important. Zoom not so good with this - miss F2F.

Reference 2 - 1.33% Coverage

But Zoom reviews can be quicker.

Reference 3 - 1.33% Coverage

Zoom calls - say as little as possible. F2F easier.

Reference 4 - 1.33% Coverage

Researchers give better responses on Zoom. It is easier to attend, more chatty and less nervous than F2F.

Reference 5 - 1.33% Coverage

Advantage of Zoom - Researchers are almost always present.
