# Supplementary material for: Evidence from UK Research Ethics Committee members on what makes a good research ethics review, and what can be improved
Source: PLoS One. 2023 Jul 3;18(7):e0288083. doi: 10.1371/journal.pone.0288083 (PMC10317218; doi:10.1371/journal.pone.0288083)
Supplement: S1 Data — (ZIP) [file pone.0288083.s001.zip › Supplementary Data/Question 1/Importance of Discussion.docx]

Files\\Qu1 - § 23 references coded [ 29.81% Coverage]

Reference 1 - 1.33% Coverage

Everyone can express their view and there is a respectful debate.

Reference 2 - 1.33% Coverage

Importance of being supportive of new members.

Reference 3 - 1.33% Coverage

Different perspectives from the committee members – learn from others.

Reference 4 - 1.33% Coverage

Risks and benefits? What are they? How are they outlined in the PIS?

Reference 5 - 1.33% Coverage

CONSENSUS

Reference 6 - 1.33% Coverage

Do we need a consensus?

Reference 7 - 1.33% Coverage

Agree on an overall view

Reference 8 - 1.33% Coverage

Mix of REC members to pick out main ethical issues

Reference 9 - 1.33% Coverage

Discussion is the key part.

Reference 10 - 1.33% Coverage

Robust process due to discussion by all members.

Reference 11 - 1.33% Coverage

Agree on questions

Reference 12 - 1.33% Coverage

NEGOTIATION

Reference 13 - 1.33% Coverage

Rarely have a vote.

Reference 14 - 1.33% Coverage

By asking questions of researchers and assessing answers.

Reference 15 - 1.33% Coverage

Useful direct questions to the applicant.

Reference 16 - 1.33% Coverage

Interview with the researcher gives a good sense.

Reference 17 - 1.33% Coverage

Lay members are very valuable because they can ask relevant questions.

Reference 18 - 1.33% Coverage

Lots of Provisional Opinions as changes are required.

Reference 19 - 1.30% Coverage

Volunteering on different RECs gives you a good idea of how to do things differently.

Reference 20 - 0.63% Coverage

we need to be sensitive to lay members

Reference 21 - 1.30% Coverage

debate, clarification, read materials, focus on IRASPIS/ICF, study synopsis, scientific value

Reference 22 - 1.30% Coverage

start off with the assumption the study is ok until you find something wrong with it.

Reference 23 - 1.29% Coverage

thorough discussion btwn all members - nobody can spot everything.
