# Supplementary material for: Evidence from UK Research Ethics Committee members on what makes a good research ethics review, and what can be improved
Source: PLoS One. 2023 Jul 3;18(7):e0288083. doi: 10.1371/journal.pone.0288083 (PMC10317218; doi:10.1371/journal.pone.0288083)
Supplement: S1 Data — (ZIP) [file pone.0288083.s001.zip › Supplementary Data/Question 1/Lead Reviewer.docx]

Files\\Qu1 - § 6 references coded [ 7.92% Coverage]

Reference 1 - 1.33% Coverage

Lead/2nd reviewer plus other member input

Reference 2 - 1.33% Coverage

Lead reviewer presents

Reference 3 - 1.33% Coverage

Process - Lead/2nd review - lead discussion, agree questions to ask, REC opinion based of pre/post researcher discussion

Reference 4 - 1.33% Coverage

LRF most useful when not the lead/2nd reviewer to pick out main ethical issues

Reference 5 - 1.31% Coverage

Follow the process - lead/second reviewer discussion, invite in, ask questions and come to an opinion after that. Some committees put on HARP.

Reference 6 - 1.27% Coverage

Broad discussion of the whole REC/Lead reviewer
