# Supplementary material for: Evidence from UK Research Ethics Committee members on what makes a good research ethics review, and what can be improved
Source: PLoS One. 2023 Jul 3;18(7):e0288083. doi: 10.1371/journal.pone.0288083 (PMC10317218; doi:10.1371/journal.pone.0288083)
Supplement: S1 Data — (ZIP) [file pone.0288083.s001.zip › Supplementary Data/Question 5/Reassurance from contribution of other members.docx]

Files\\Qu5 - § 4 references coded [ 8.16% Coverage]

Reference 1 - 2.04% Coverage

There is reassurance that other members spot protocol issues.

Reference 2 - 2.04% Coverage

REC discussion is reassuring to make sure that nothing is being missed.

Reference 3 - 2.04% Coverage

Wide range of opinions between committees builds confidence in the REC’s ability.

Reference 4 - 2.04% Coverage

Nurturing environments.
