# Supplementary material for: Evidence from UK Research Ethics Committee members on what makes a good research ethics review, and what can be improved
Source: PLoS One. 2023 Jul 3;18(7):e0288083. doi: 10.1371/journal.pone.0288083 (PMC10317218; doi:10.1371/journal.pone.0288083)
Supplement: S1 Data — (ZIP) [file pone.0288083.s001.zip › Supplementary Data/Question 5/Feedback from stakeholders.docx]

Files\\Qu5 - § 15 references coded [ 30.11% Coverage]

Reference 1 - 2.04% Coverage

Satisfied doing a good job: Researcher feedback, HRA feedback, participant complaints (evidence maybe things went wrong) maybe ask for participant feedback for both positive and negative comments).

Reference 2 - 2.04% Coverage

Chairs receive any feedback that can be linked to their committee, but if the REC could have more regular feedback from applicants would be really helpful.

Reference 3 - 2.04% Coverage

Feedback and receiving quality research following REC review provides satisfaction.

Reference 4 - 2.04% Coverage

The opportunity to give feedback (from the applicant??) via the feedback link should be really prominent.

Reference 5 - 2.04% Coverage

Good job - we get thanks for Researchers and we feel we are part of the journey to good research.

Reference 6 - 2.04% Coverage

UK RECs more collaborative than REC abroad who are more combative and hostile.

Reference 7 - 2.04% Coverage

Researchers happy to do the research and thank REC

Reference 8 - 2.04% Coverage

The feedback from the researchers.

Reference 9 - 1.94% Coverage

getting positive feedback from the applicant.

Reference 10 - 1.94% Coverage

positive feedback and a sense of resolution.

Reference 11 - 2.01% Coverage

The table agreed that it would be useful for individual RECs to receive feedback, although this does happen sometimes, they would like to receive more feedback.

Reference 12 - 1.97% Coverage

Feedback from applicants. Some chairs share the overall comments.

Reference 13 - 1.93% Coverage

Feedback of nice comments not sufficient

Reference 14 - 2.00% Coverage

the research actually happens, appropriate lack of harm to participants, feedback from the applicant.

Reference 15 - 1.99% Coverage

positive feedback from the applicant is validating and shows that what we are doing is important.
