# Supplementary material for: Evidence from UK Research Ethics Committee members on what makes a good research ethics review, and what can be improved
Source: PLoS One. 2023 Jul 3;18(7):e0288083. doi: 10.1371/journal.pone.0288083 (PMC10317218; doi:10.1371/journal.pone.0288083)
Supplement: S1 Data — (ZIP) [file pone.0288083.s001.zip › Supplementary Data/Question 5/Confidence from talking to researchers.docx]

Files\\Qu5 - § 3 references coded [ 5.94% Coverage]

Reference 1 - 2.04% Coverage

Asking applicants questions builds confidence and satisfaction of input.

Reference 2 - 1.98% Coverage

Constructive engagement with the applicant, can be satisfied good job in depth.

Reference 3 - 1.92% Coverage

when +ve interaction or -ve you know.
