# Supplementary material for: Evidence from UK Research Ethics Committee members on what makes a good research ethics review, and what can be improved
Source: PLoS One. 2023 Jul 3;18(7):e0288083. doi: 10.1371/journal.pone.0288083 (PMC10317218; doi:10.1371/journal.pone.0288083)
Supplement: S1 Data — (ZIP) [file pone.0288083.s001.zip › Supplementary Data/Question 5/Timely & Efficient process.docx]

Files\\Qu5 - § 4 references coded [ 8.16% Coverage]

Reference 1 - 2.04% Coverage

REC time management. When the agenda/timetable is being adhered to there is a confidence that the reviews are efficient and that there have not been major concerns with studies. A question was raised – how long does a study need on average? 45 minutes? An hour?

Reference 2 - 2.04% Coverage

Can sometimes feel haven’t done a good job if time pressured i.e. less time on a particular study.

Reference 3 - 2.04% Coverage

REC Chair Anxiety? Does the Chair get anxious after the decision when the letter goes out? No – usually content as a Chair because the process has been good (Chris….?)

Reference 4 - 2.04% Coverage

A lunch break!
