# Supplementary material for: Evidence from UK Research Ethics Committee members on what makes a good research ethics review, and what can be improved
Source: PLoS One. 2023 Jul 3;18(7):e0288083. doi: 10.1371/journal.pone.0288083 (PMC10317218; doi:10.1371/journal.pone.0288083)
Supplement: S1 Data — (ZIP) [file pone.0288083.s001.zip › Supplementary Data/Question 5/Seeing outcomes of research.docx]

Files\\Qu5 - § 5 references coded [ 10.14% Coverage]

Reference 1 - 2.04% Coverage

Feedback and receiving quality research following REC review provides satisfaction.

Reference 2 - 2.04% Coverage

UK produces more good quality research than anywhere else.

Reference 3 - 2.04% Coverage

Research has improved

Reference 4 - 2.04% Coverage

Contribution to the research process

Reference 5 - 1.97% Coverage

he research actually happens, appropriate lack of harm to participants, feedback from the applicant.
