# Supplementary material for: Evidence from UK Research Ethics Committee members on what makes a good research ethics review, and what can be improved
Source: PLoS One. 2023 Jul 3;18(7):e0288083. doi: 10.1371/journal.pone.0288083 (PMC10317218; doi:10.1371/journal.pone.0288083)
Supplement: S1 Data — (ZIP) [file pone.0288083.s001.zip › Supplementary Data/Question 5/Duplicate.docx]

Files\\Qu5 - § 5 references coded [ 10.20% Coverage]

Reference 1 - 2.04% Coverage

There is reassurance that other members spot protocol issues.

Reference 2 - 2.04% Coverage

HRA Safety net? If something is missed, will the HRA catch this as a backup? No – the HRA role is not to do this, but to cover governance and management of the process. But… what bad things have ever happened due to REC missing something? Even TGN1412 showed that there were fundamental issues way beyond the remit of REC review.

Reference 3 - 2.04% Coverage

REC time management. When the agenda/timetable is being adhered to there is a confidence that the reviews are efficient and that there have not been major concerns with studies. A question was raised – how long does a study need on average? 45 minutes? An hour?

Reference 4 - 2.04% Coverage

The system is not broken. The RECs have reviewed 1000s of studies. There have been few if any disasters post REC review. There is always scope to reflect and improve the reviews, but the system is not broken and does not need to be replaced.

Reference 5 - 2.04% Coverage

We’re doing a good job - it has been many years since any major research scandal.
