# Supplementary material for: Evidence from UK Research Ethics Committee members on what makes a good research ethics review, and what can be improved
Source: PLoS One. 2023 Jul 3;18(7):e0288083. doi: 10.1371/journal.pone.0288083 (PMC10317218; doi:10.1371/journal.pone.0288083)
Supplement: S1 Data — (ZIP) [file pone.0288083.s001.zip › Supplementary Data/Question 5/Committee reflection.docx]

Files\\Qu5 - § 5 references coded [ 9.94% Coverage]

Reference 1 - 1.99% Coverage

at the end of the REC meeting, so a wash up sessions - did we do a good job or do we feel weary?

Reference 2 - 1.97% Coverage

was it a good interaction with the applicant, was there good dialogue?

Reference 3 - 1.97% Coverage

chair feedback on a good review is important (also for new REC members).

Reference 4 - 2.01% Coverage

At the end of every meeting the REC could ask themselves ‘what have we done to improve the quality of the research presented to us today’

Reference 5 - 2.00% Coverage

feel we have done a good job when we know a participant will understand what they have/are being asked to do by taking part.
