# Supplementary material for: Evidence from UK Research Ethics Committee members on what makes a good research ethics review, and what can be improved
Source: PLoS One. 2023 Jul 3;18(7):e0288083. doi: 10.1371/journal.pone.0288083 (PMC10317218; doi:10.1371/journal.pone.0288083)
Supplement: S1 Data — (ZIP) [file pone.0288083.s001.zip › Supplementary Data/Question 5/Comparison with other RECs.docx]

Files\\Qu5 - § 3 references coded [ 6.09% Coverage]

Reference 1 - 2.04% Coverage

Is there any kind of peer review for ethics committees?

Reference 2 - 2.04% Coverage

UK RECs more collaborative than REC abroad who are more combative and hostile.

Reference 3 - 2.00% Coverage

Often a PO - the comments come back to the Chair. But if everybody saw those it would give a better idea if a good job (if contentious)
