# Supplementary material for: Evidence from UK Research Ethics Committee members on what makes a good research ethics review, and what can be improved
Source: PLoS One. 2023 Jul 3;18(7):e0288083. doi: 10.1371/journal.pone.0288083 (PMC10317218; doi:10.1371/journal.pone.0288083)
Supplement: S1 Data — (ZIP) [file pone.0288083.s001.zip › Supplementary Data/Question 2/Participants Perspective.docx]

Files\\Qu2 - § 5 references coded [ 7.52% Coverage]

Reference 1 - 1.59% Coverage

Being the voice of the patient/participant – having their perspective.

Reference 2 - 1.59% Coverage

PPI

Reference 3 - 1.55% Coverage

Social care Homes - a presumption that dining/living rooms are public spaces instead of people’s homes.

Reference 4 - 1.56% Coverage

an example of insensitive research - collection of ova at caesarean sections, because it was easier to collect that that stage

Reference 5 - 1.23% Coverage

good PPI
