# Supplementary material for: Evidence from UK Research Ethics Committee members on what makes a good research ethics review, and what can be improved
Source: PLoS One. 2023 Jul 3;18(7):e0288083. doi: 10.1371/journal.pone.0288083 (PMC10317218; doi:10.1371/journal.pone.0288083)
Supplement: S1 Data — (ZIP) [file pone.0288083.s001.zip › Supplementary Data/Question 2/Different Perspectives.docx]

Files\\Qu2 - § 3 references coded [ 4.76% Coverage]

Reference 1 - 1.59% Coverage

The broad range of views and opinions on the REC, the group variety makes for good ethical decisions. You look at studies from many different angles. For example, a lay member could ask an obvious question that has not been asked or assumed.

Reference 2 - 1.59% Coverage

Would be good to have experts in different fields on each REC (e.g. pharmacist for a CTIMP)

Reference 3 - 1.59% Coverage

Discuss Radiation/Pharmacy assurance.
