# Supplementary material for: Evidence from UK Research Ethics Committee members on what makes a good research ethics review, and what can be improved
Source: PLoS One. 2023 Jul 3;18(7):e0288083. doi: 10.1371/journal.pone.0288083 (PMC10317218; doi:10.1371/journal.pone.0288083)
Supplement: S1 Data — (ZIP) [file pone.0288083.s001.zip › Supplementary Data/Question 2/Participant Information & Consent.docx]

Files\\Qu2 - § 9 references coded [ 14.03% Coverage]

Reference 1 - 1.59% Coverage

Key questions: is it clear to the participant what is going on? What are the risks? Is there consistency across all documents?

Reference 2 - 1.59% Coverage

Look at specific issues withing study types - e.g. ALC studies have different PIS issues.

Reference 3 - 1.59% Coverage

Consent questions - understanding the context of the consent process

Reference 4 - 1.59% Coverage

PIS etc - making sure they are relevant and satisfactory.

Reference 5 - 1.59% Coverage

Is the PIS good enough to make an informed decision.

Reference 6 - 1.52% Coverage

make sure the PIS is easy to read and is short enough

Reference 7 - 1.50% Coverage

is equipoise understood and explained?

Reference 8 - 1.54% Coverage

Patient safety, Cohesion, PIS is very important, MCA concerns and PPI

Reference 9 - 1.55% Coverage

scientific/clinical value, clarify of pt focussed documents, PPI, risk-benefit, equipoise
