# Supplementary material for: Evidence from UK Research Ethics Committee members on what makes a good research ethics review, and what can be improved
Source: PLoS One. 2023 Jul 3;18(7):e0288083. doi: 10.1371/journal.pone.0288083 (PMC10317218; doi:10.1371/journal.pone.0288083)
Supplement: S1 Data — (ZIP) [file pone.0288083.s001.zip › Supplementary Data/Question 2/Consistency of Review Process.docx]

Files\\Qu2 - § 7 references coded [ 10.92% Coverage]

Reference 1 - 1.59% Coverage

Subjective – REC members can sometimes focus on issues other people consider peripheral, so it’s important that the REC discuss and feel clear on the types of issues to consider.

Reference 2 - 1.59% Coverage

Differentiate between questions that are asked in person or points that can just be put in the letter.

Reference 3 - 1.59% Coverage

Protocol. Some concern if all REC members are advised not to read the protocol fully – as there are often inconsistencies or issues that can remain unidentified. The protocol is then being approved by the REC without full knowledge – introduces a risk.

Reference 4 - 1.59% Coverage

UFO – how does the REC communicate effectively when a PhD student has received a UFO and the supervisor was not in the REC meeting?

Reference 5 - 1.52% Coverage

focus on the major ethical issues, the key questions

Reference 6 - 1.53% Coverage

Focus on the major issues, the fundamentals not the smaller points

Reference 7 - 1.52% Coverage

look at fundamental versus less fundamental issues.
