# Supplementary material for: Evidence from UK Research Ethics Committee members on what makes a good research ethics review, and what can be improved
Source: PLoS One. 2023 Jul 3;18(7):e0288083. doi: 10.1371/journal.pone.0288083 (PMC10317218; doi:10.1371/journal.pone.0288083)
Supplement: S1 Data — (ZIP) [file pone.0288083.s001.zip › Supplementary Data/Question 2/Researcher's attitude & experience.docx]

Files\\Qu2 - § 5 references coded [ 7.86% Coverage]

Reference 1 - 1.59% Coverage

Respect for the patient both in documents and researcher attitude in the meeting.

Reference 2 - 1.59% Coverage

Having the researcher present to answer questions is very helpful.

Reference 3 - 1.59% Coverage

Ask the researchers to explain the study to REC and see how it will sound to participants.

Reference 4 - 1.54% Coverage

"No ethical issues" is a red flag - the applicant hasn’t thought the study through

Reference 5 - 1.56% Coverage

applications coming in from people who have no experience in health or social care and don’t understand the impact of what they are doing.
