# Supplementary material for: Evidence from UK Research Ethics Committee members on what makes a good research ethics review, and what can be improved
Source: PLoS One. 2023 Jul 3;18(7):e0288083. doi: 10.1371/journal.pone.0288083 (PMC10317218; doi:10.1371/journal.pone.0288083)
Supplement: S1 Data — (ZIP) [file pone.0288083.s001.zip › Supplementary Data/Question 2/Risk & Safety.docx]

Files\\Qu2 - § 19 references coded [ 29.85% Coverage]

Reference 1 - 1.59% Coverage

Do we look at ethics or safeguards?

Reference 2 - 1.59% Coverage

Key questions: is it clear to the participant what is going on? What are the risks? Is there consistency across all documents?

Reference 3 - 1.59% Coverage

Safety very important

Reference 4 - 1.59% Coverage

Potential for harm/distress

Reference 5 - 1.59% Coverage

Harm to patients.

Reference 6 - 1.59% Coverage

Care and safeguarding - making sure the patients are not dumped after the study.

Reference 7 - 1.59% Coverage

Risk / benefit

Reference 8 - 1.59% Coverage

Patient safety

Reference 9 - 1.59% Coverage

Balance Risk/Benefit

Reference 10 - 1.59% Coverage

Risks/Benefits ratio

Reference 11 - 1.59% Coverage

Researchers who know how to mitigate risk.

Reference 12 - 1.59% Coverage

Burden.

Reference 13 - 1.59% Coverage

The follow up.

Reference 14 - 1.59% Coverage

Questions about mental health and then nothing afterwards.

Reference 15 - 1.59% Coverage

Columbia scale re depression/suicide - only when this is not self-administered.

Reference 16 - 1.53% Coverage

Key issues? Protect the patients, or if the study is not good quality

Reference 17 - 1.54% Coverage

the MCA is often misunderstood and safeguarding issues related to it are easy to miss

Reference 18 - 1.43% Coverage

Risk/benefit issues

Reference 19 - 1.54% Coverage

Patient safety, Cohesion, PIS is very important, MCA concerns and PPI
