# Supplementary material for: Evidence from UK Research Ethics Committee members on what makes a good research ethics review, and what can be improved
Source: PLoS One. 2023 Jul 3;18(7):e0288083. doi: 10.1371/journal.pone.0288083 (PMC10317218; doi:10.1371/journal.pone.0288083)
Supplement: S1 Data — (ZIP) [file pone.0288083.s001.zip › Supplementary Data/Question 2/Research Design & Justification.docx]

Files\\Qu2 - § 11 references coded [ 17.05% Coverage]

Reference 1 - 1.59% Coverage

Is poor research design a material ethical issue?

Reference 2 - 1.59% Coverage

Is it worth doing the study - got to have benefits

Reference 3 - 1.59% Coverage

Is it worth doing?

Reference 4 - 1.59% Coverage

Study Design.

Reference 5 - 1.59% Coverage

We can assist in improving the study design by giving written feedback to the researchers which they can show to the sponsors.

Reference 6 - 1.47% Coverage

Scientific validity is vital

Reference 7 - 1.53% Coverage

how much science to do scrutinise, how much is the MHRA concern?

Reference 8 - 1.49% Coverage

Understanding is the fundamental aim

Reference 9 - 1.51% Coverage

will the proposal lead to the proposed outcome?

Reference 10 - 1.55% Coverage

scientific/clinical value, clarify of pt focussed documents, PPI, risk-benefit, equipoise

Reference 11 - 1.56% Coverage

does the study answer the research question - fundamental. Doing something that isn’t going to produce a valid good result is pointless.
