# Supplementary material for: Evidence from UK Research Ethics Committee members on what makes a good research ethics review, and what can be improved
Source: PLoS One. 2023 Jul 3;18(7):e0288083. doi: 10.1371/journal.pone.0288083 (PMC10317218; doi:10.1371/journal.pone.0288083)
Supplement: S1 Data — (ZIP) [file pone.0288083.s001.zip › Supplementary Data/Question 2/Consistency & Clarity of Information.docx]

Files\\Qu2 - § 6 references coded [ 9.37% Coverage]

Reference 1 - 1.59% Coverage

Key questions: is it clear to the participant what is going on? What are the risks? Is there consistency across all documents?

Reference 2 - 1.59% Coverage

Can I understand what the study is about? What are they doing? This is key before you continue the review.

Reference 3 - 1.59% Coverage

Can I understand the project?

Reference 4 - 1.59% Coverage

The most ethical questions based on the study in front of you.

Reference 5 - 1.55% Coverage

can we understand the protocol/IRAS form? was the applicant just not good at filling in forms or is it a poor study?

Reference 6 - 1.47% Coverage

is the information complete?
