# Supplementary material for: Evidence from UK Research Ethics Committee members on what makes a good research ethics review, and what can be improved
Source: PLoS One. 2023 Jul 3;18(7):e0288083. doi: 10.1371/journal.pone.0288083 (PMC10317218; doi:10.1371/journal.pone.0288083)
Supplement: S1 Data — (ZIP) [file pone.0288083.s001.zip › Supplementary Data/Question 3/Structures or Shortens review.docx]

Files\\Qu3 - § 18 references coded [ 30.49% Coverage]

Reference 1 - 1.66% Coverage

SK asked if the LRF lengthens the REC review? Newer REC members really like it. The LRF gives structure and shortens reviews.

Reference 2 - 1.72% Coverage

LRF. Mixed use, but those who do use it find it helpful to focus on important points.

Reference 3 - 1.65% Coverage

Chairs find it useful, but is it really ethics? Is it good for the patient is a key question. Thus is key to ethics.

Reference 4 - 1.72% Coverage

To organise in sequential order your review based on ethical domains

Reference 5 - 1.72% Coverage

Guidance for review

Reference 6 - 1.72% Coverage

Helpful for REC discussion

Reference 7 - 1.72% Coverage

To improve the research.

Reference 8 - 1.72% Coverage

To summarise the key points.

Reference 9 - 1.72% Coverage

To make the decisions more clear.

Reference 10 - 1.72% Coverage

Helpful for REC Opinion.

Reference 11 - 1.72% Coverage

The LRF helps the REC keep to time.

Reference 12 - 1.68% Coverage

ERF is great for ALC studies and helps with CWOW too (the CWOW IRAS form is not easy to use)

Reference 13 - 1.64% Coverage

the ERF encourages everybody to contribute.

Reference 14 - 1.69% Coverage

ERF - It can improve the minutes as if the minute takes misses a point at the meeting they can refer to the ethics review form.

Reference 15 - 1.70% Coverage

ERF - Issues with the PIS and other documents do not need to be raised during the meeting they can be detailed on the form and added to the letter. Saving time at the meeting.

Reference 16 - 1.68% Coverage

ERF - a good way to structure the conversation. It’s important to raise the with issues. Consistency.

Reference 17 - 1.64% Coverage

ERF - there to give focus, provide a checklist

Reference 18 - 1.63% Coverage

ERF - ensure the review form is inclusive
