# Supplementary material for: Evidence from UK Research Ethics Committee members on what makes a good research ethics review, and what can be improved
Source: PLoS One. 2023 Jul 3;18(7):e0288083. doi: 10.1371/journal.pone.0288083 (PMC10317218; doi:10.1371/journal.pone.0288083)
Supplement: S1 Data — (ZIP) [file pone.0288083.s001.zip › Supplementary Data/Question 3/Wrong topics.docx]

Files\\Qu3 - § 5 references coded [ 8.46% Coverage]

Reference 1 - 1.72% Coverage

LRF not really ethics issues, more Governance.

Reference 2 - 1.65% Coverage

Chairs find it useful, but is it really ethics? Is it good for the patient is a key question. Thus is key to ethics.

Reference 3 - 1.72% Coverage

Useful design but not guidance for ethics - this is the REC remit.

Reference 4 - 1.72% Coverage

Needs to focus on ’ethics’

Reference 5 - 1.64% Coverage

ERF - difficult to box ethical issues though
