# Supplementary material for: Evidence from UK Research Ethics Committee members on what makes a good research ethics review, and what can be improved
Source: PLoS One. 2023 Jul 3;18(7):e0288083. doi: 10.1371/journal.pone.0288083 (PMC10317218; doi:10.1371/journal.pone.0288083)
Supplement: S1 Data — (ZIP) [file pone.0288083.s001.zip › Supplementary Data/Question 3/Conflict between documents.docx]

Files\\Qu3 - § 7 references coded [ 12.02% Coverage]

Reference 1 - 1.72% Coverage

LRF versus IRAS form versus Protocol. They are not intuitive and there is a mismatch/misalignment.

Reference 2 - 1.72% Coverage

LRF – there are different versions (do some mention insurance which is an HRA issue??) MCA, CTIMP different versions.

Reference 3 - 1.72% Coverage

LRF – this would be helpful if it aligned with the order of the questions on the IRAS form.

Reference 4 - 1.67% Coverage

When asked in the session, 80%+ said they use the LRF form and found it helpful. [NOTE there have been different versions and now it is the LRF on HARP].

Reference 5 - 1.72% Coverage

LRF form does not match CWOW.

Reference 6 - 1.72% Coverage

LRF form easy to use? There are often too many entries and it is clunky. It would be better if the sections could be opened and closed more easily.

Reference 7 - 1.72% Coverage

Not always useful - e.g. CWOW.
