# Supplementary material for: Evidence from UK Research Ethics Committee members on what makes a good research ethics review, and what can be improved
Source: PLoS One. 2023 Jul 3;18(7):e0288083. doi: 10.1371/journal.pone.0288083 (PMC10317218; doi:10.1371/journal.pone.0288083)
Supplement: S1 Data — (ZIP) [file pone.0288083.s001.zip › Supplementary Data/Question 3/New section.docx]

Files\\Qu3 - § 2 references coded [ 3.40% Coverage]

Reference 1 - 1.72% Coverage

Add a box for "what questions do you want to ask?"

Reference 2 - 1.68% Coverage

ERF - SK commented that the paper version of the ERF was better - section 5 for key REC issues.
