# Supplementary material for: Evidence from UK Research Ethics Committee members on what makes a good research ethics review, and what can be improved
Source: PLoS One. 2023 Jul 3;18(7):e0288083. doi: 10.1371/journal.pone.0288083 (PMC10317218; doi:10.1371/journal.pone.0288083)
Supplement: S1 Data — (ZIP) [file pone.0288083.s001.zip › Supplementary Data/Question 3/Duplicate.docx]

Files\\Qu3 - § 5 references coded [ 8.62% Coverage]

Reference 1 - 1.72% Coverage

LRF. This is used to communicate with other members before the meeting and stimulate discussion. [NOTE – the HRA Approval Specialists like this, it helps prepare for the meeting and reduce discussion of Governance issues].

Reference 2 - 1.72% Coverage

LRF: SK asked if the LRF lengthens the REC review? Newer REC members really like it. The LRF gives structure and shortens reviews.

Reference 3 - 1.72% Coverage

LRF: are the right questions asked? Yes, they are topics areas rather than questions and they cover the important areas.

Reference 4 - 1.72% Coverage

LRF versus IRAS form versus Protocol. They are not intuitive and there is a mismatch/misalignment.

Reference 5 - 1.72% Coverage

LRF – there are different versions (do some mention insurance which is an HRA issue??) MCA, CTIMP different versions.
