# Supplementary material for: Evidence from UK Research Ethics Committee members on what makes a good research ethics review, and what can be improved
Source: PLoS One. 2023 Jul 3;18(7):e0288083. doi: 10.1371/journal.pone.0288083 (PMC10317218; doi:10.1371/journal.pone.0288083)
Supplement: S1 Data — (ZIP) [file pone.0288083.s001.zip › Supplementary Data/Question 3/Prepare for meeting.docx]

Files\\Qu3 - § 11 references coded [ 18.40% Coverage]

Reference 1 - 1.69% Coverage

This is used to communicate with other members before the meeting and stimulate discussion. [NOTE – the HRA Approval Specialists like this, it helps prepare for the meeting and reduce discussion of Governance issues].

Reference 2 - 1.57% Coverage

Useful for Chairs and Lead reviewers especially.

Reference 3 - 1.72% Coverage

LRF – HARP portal – sharing comments here pre-meeting helps avoid duplication – exception reporting.

Reference 4 - 1.72% Coverage

LRF. Share this prior to the meeting as part of preparatory work.

Reference 5 - 1.72% Coverage

Add a box for "what questions do you want to ask?"

Reference 6 - 1.65% Coverage

ERF - useful to share comments before the meeting.

Reference 7 - 1.68% Coverage

ERF - the lead/Second reviewer can collaborate collate thoughts before the meeting.

Reference 8 - 1.68% Coverage

ERF - make sure it is completed in time for other people to read and review and express disagreements.

Reference 9 - 1.67% Coverage

ERF - proves very useful. Able to look at other members thoughts and comments.

Reference 10 - 1.61% Coverage

ERF - able to see things earlier.

Reference 11 - 1.68% Coverage

ERF - lead/second reviewers often collate comments so they can be discussed in the meeting
