# Supplementary material for: Evidence from UK Research Ethics Committee members on what makes a good research ethics review, and what can be improved
Source: PLoS One. 2023 Jul 3;18(7):e0288083. doi: 10.1371/journal.pone.0288083 (PMC10317218; doi:10.1371/journal.pone.0288083)
Supplement: S1 Data — (ZIP) [file pone.0288083.s001.zip › Supplementary Data/Question 3/Right topics.docx]

Files\\Qu3 - § 3 references coded [ 4.95% Coverage]

Reference 1 - 1.66% Coverage

are the right questions asked? Yes, they are topics areas rather than questions and they cover the important areas.

Reference 2 - 1.66% Coverage

ERF - a good way to summarise discussions, especially CWOW.

Reference 3 - 1.64% Coverage

ERF - All the issues to be raised are in one place
