# Supplementary material for: Evidence from UK Research Ethics Committee members on what makes a good research ethics review, and what can be improved
Source: PLoS One. 2023 Jul 3;18(7):e0288083. doi: 10.1371/journal.pone.0288083 (PMC10317218; doi:10.1371/journal.pone.0288083)
Supplement: S1 Data — (ZIP) [file pone.0288083.s001.zip › Supplementary Data/Question 3/Not everyone uses it.docx]

Files\\Qu3 - § 8 references coded [ 13.51% Coverage]

Reference 1 - 1.72% Coverage

LRF – some use it, some don’t.

Reference 2 - 1.72% Coverage

Lead reviewer Form (LRF). Yes, we use this a lot. Not so much the longer serving members.

Reference 3 - 1.72% Coverage

LRF - used on Fast Track. It helped focus. Most ppl use the LRF. Very few don’t - usually the more experienced members don’t.

Reference 4 - 1.67% Coverage

When asked in the session, 80%+ said they use the LRF form and found it helpful. [NOTE there have been different versions and now it is the LRF on HARP].

Reference 5 - 1.72% Coverage

Some places not used [RECs or sections??]

Reference 6 - 1.64% Coverage

Use the ERF - some Chairs say this is compulsory.

Reference 7 - 1.62% Coverage

ERF - some use it some use it some don’t.

Reference 8 - 1.68% Coverage

ERF - we asked directly - and about 80% of people say they use the ERF, mostly newer REC volunteers.
