# Supplementary material for: Evidence from UK Research Ethics Committee members on what makes a good research ethics review, and what can be improved
Source: PLoS One. 2023 Jul 3;18(7):e0288083. doi: 10.1371/journal.pone.0288083 (PMC10317218; doi:10.1371/journal.pone.0288083)
Supplement: S1 Data — (ZIP) [file pone.0288083.s001.zip › Supplementary Data/Question 4/Structure of discussion.docx]

Files\\Qu4 - § 7 references coded [ 29.37% Coverage]

Reference 1 - 4.55% Coverage

After an appropriate discussion.

Reference 2 - 4.55% Coverage

Who asks the questions? The Chair or Lead/2nd reviewer. Or other members.

Reference 3 - 4.43% Coverage

Full REC discussion or Lead/second reviewer discussion led. Or all agree on weighty issues.

Reference 4 - 4.38% Coverage

the lead reviewer carries the weight of focussing the review.

Reference 5 - 2.73% Coverage

Yes

Reference 6 - 4.28% Coverage

no uniform standard, should there be?

Reference 7 - 4.47% Coverage

some applicants are more receptive to issues raised than others are - how applicants respond can have an impact on the opinion the REC give.
