# Supplementary material for: Evidence from UK Research Ethics Committee members on what makes a good research ethics review, and what can be improved
Source: PLoS One. 2023 Jul 3;18(7):e0288083. doi: 10.1371/journal.pone.0288083 (PMC10317218; doi:10.1371/journal.pone.0288083)
Supplement: S1 Data — (ZIP) [file pone.0288083.s001.zip › Supplementary Data/Question 4/Co-opting members.docx]

Files\\Qu4 - § 1 reference coded [ 4.55% Coverage]

Reference 1 - 4.55% Coverage

Co-opting. This is an important thing to do to see how other RECs operate and get some insight.
