# Supplementary material for: Evidence from UK Research Ethics Committee members on what makes a good research ethics review, and what can be improved
Source: PLoS One. 2023 Jul 3;18(7):e0288083. doi: 10.1371/journal.pone.0288083 (PMC10317218; doi:10.1371/journal.pone.0288083)
Supplement: S1 Data — (ZIP) [file pone.0288083.s001.zip › Supplementary Data/Question 4/Role of Chair.docx]

Files\\Qu4 - § 4 references coded [ 17.88% Coverage]

Reference 1 - 4.55% Coverage

Chair decides and persuades other REC members.

Reference 2 - 4.55% Coverage

Who asks the questions? The Chair or Lead/2nd reviewer. Or other members.

Reference 3 - 4.36% Coverage

different chairs, different methods. Chairs ask, tell, lead reviewer does this, group discussion and occasionally voting if it comes to it.

Reference 4 - 4.43% Coverage

Some Chairs ask the REC, some use the Lead/Second reviewer. Some have a group discussion.
