# Supplementary material for: Evidence from UK Research Ethics Committee members on what makes a good research ethics review, and what can be improved
Source: PLoS One. 2023 Jul 3;18(7):e0288083. doi: 10.1371/journal.pone.0288083 (PMC10317218; doi:10.1371/journal.pone.0288083)
Supplement: S1 Data — (ZIP) [file pone.0288083.s001.zip › Supplementary Data/Question 4/Different approach to documents.docx]

Files\\Qu4 - § 7 references coded [ 31.04% Coverage]

Reference 1 - 4.55% Coverage

Protocol – review as needed.

Reference 2 - 4.55% Coverage

Some people start with the PIS (as if they were a participant), rather than the protocol.

Reference 3 - 4.48% Coverage

ShED minutes analysis showed that some RECs reach the top 10 key issues succinctly and quickly. Others by discussing many more issues, almost a scattergun approach. So, there is clearly a difference in approaches and character.

Reference 4 - 4.40% Coverage

Choose - focus on the main issues in the application or scrutinise it all.

Reference 5 - 4.23% Coverage

compare the PIS and the protocol

Reference 6 - 4.39% Coverage

PIS - review is from both the applicant and participant perspective.

Reference 7 - 4.45% Coverage

There is an assumption that everybody has read everything and it’s a group decision, but is this always the case?
