# Supplementary material for: Evidence from UK Research Ethics Committee members on what makes a good research ethics review, and what can be improved
Source: PLoS One. 2023 Jul 3;18(7):e0288083. doi: 10.1371/journal.pone.0288083 (PMC10317218; doi:10.1371/journal.pone.0288083)
Supplement: Attachment — Submitted filename: Response to Reviewers.docx [file pone.0288083.s002.docx]

17^th^ April 2023

Dear Dr Lennox,

Thank you for arranging the review of our manuscript:

***PONE-D-23-04580 Evidence from UK Research Ethics Committee members on what makes a good research ethics review, and what can be improved.***

Peer review of a manuscript can sometime be a frustrating process, however in this case we were really appreciative of the constructive comments as they helped us to think carefully about this manuscript and what we are trying to present.

For some context, it is an interesting facet of research ethics review that committees are coordinated by administrative and/or regulatory authorities whose main aims is policy compliance and efficiency rather than academic transparency in the form of publications. As a consequence, despite the significant amount of work that goes into improving processes, the results are seldom released publicly as they instead take the form of updated standard operating procedures, guidance etc. that are seldom communicated in any meaningful way outside the organisation involved. This is a real weakness in the research system because organisations such as the UK’s Health Research Authority have considerable experience in research ethics review that is not well communicated.

To address this the aim of this paper was to take a really interesting piece of work conducted by the HRA as a service improvement activity, and try to make it more available for the wider research ethics community. The work was therefore not originally designed as a research project, and thus certain components – such as holding independent focus groups that could be recorded and transcribed – were not possible. However, despite these limitations, our aim was to turn what might have otherwise been a purely administrative (and potentially soon forgotten) project into a robust data collection and analysis that will hopefully meet the standard, and be of interest, to the literature and thus wider community.

The following describes our response to the review with *reviewers comments in italic*, and our comments marked in green (on submission we were asked to upload an unformatted version of this letter, but we note a version that retains the colours was included at the bottom of the pdf file).

*Journal Requirements:*

*When submitting your revision, we need you to address these additional requirements.*

*1. Please ensure that your manuscript meets PLOS ONE's style requirements, including those for file naming. The PLOS ONE style templates can be found at*

*https://journals.plos.org/plosone/s/file?id=wjVg/PLOSOne_formatting_sample_main_body.pdf and*

*https://journals.plos.org/plosone/s/file?id=ba62/PLOSOne_formatting_sample_title_authors_affiliations.pdf*

This has been done.

*2. Thank you for stating the following financial disclosure:*

*"The work described here was conducted as part of the normal duties of MS and CC as part of their employment with the Health Research Authority. SEK was seconded to the Health Research Authority to conduct this work, and was remunerated through the University of Portsmouth."*

*Please state what role the funders took in the study. If the funders had no role, please state: "The funders had no role in study design, data collection and analysis, decision to publish, or preparation of the manuscript."*

*If this statement is not correct you must amend it as needed.*

*Please include this amended Role of Funder statement in your cover letter; we will change the online submission form on your behalf.*

The following is our revised funder statement:

“The work described here was conducted as normal duties for MS and CC as part of their employment with the Health Research Authority (HRA). SEK was seconded to the Health Research Authority to conduct this work, and was remunerated through the University of Portsmouth. As employees of the HRA this work was designed, and data collected, by the funder, however the analysis, decision to publish and preparation of the manuscript was conducted independently.”

*3. Thank you for stating the following in the Competing Interests section:*

*"MS and CC work for the UK Health Research Authority. SEK was seconded to the HRA to conduct this research, and is also chair of the Cambridgeshire and Hertfordshire Research Ethics Committee. SEK is also a trustee of the charity UKRIO."*

*Please confirm that this does not alter your adherence to all PLOS ONE policies on sharing data and materials, by including the following statement: "This does not alter our adherence to PLOS ONE policies on sharing data and materials.” (as detailed online in our guide for authors http://journals.plos.org/plosone/s/competing-interests). If there are restrictions on sharing of data and/or materials, please state these. Please note that we cannot proceed with consideration of your article until this information has been declared.*

*Please include your updated Competing Interests statement in your cover letter; we will change the online submission form on your behalf.*

We are happy to include this additional like in the Competing Interests section so that it now reads:

“MS and CC work for the UK Health Research Authority. SEK was seconded to the HRA to conduct this research, and is also chair of the Cambridgeshire and Hertfordshire Research Ethics Committee. SEK is also a trustee of the charity UKRIO. This does not alter our adherence to PLOS ONE policies on sharing data and materials.”

*4. We note that you have indicated that data from this study are available upon request. PLOS only allows data to be available upon request if there are legal or ethical restrictions on sharing data publicly. For more information on unacceptable data access restrictions, please see http://journals.plos.org/plosone/s/data-availability#loc-unacceptable-data-access-restrictions.*

*[text with various options deleted]*

We have now made our data available as supplementary information.

*5. Please include your full ethics statement in the ‘Methods’ section of your manuscript file. In your statement, please include the full name of the IRB or ethics committee who approved or waived your study, as well as whether or not you obtained informed written or verbal consent. If consent was waived for your study, please include this information in your statement as well.*

This has been done.

*6. We note you have included a table to which you do not refer in the text of your manuscript. Please ensure that you refer to Table 1,4, 6 and 7in your text; if accepted, production will need this reference to link the reader to the Table.*

This has been done.

*Additional Editor Comments:*

*Introduction*

*Line 60 - RECS should be RECs*

Corrected

*Line 67 - for a wider readership some more context is needed on 'Think Ethics', why was it established? What was the evidenced rationale for it? Is there a wider literature to support this, perhaps from other countries?*

It has been clarified that the purpose of “Think Ethics” was to act on lessons learned from the COVID pandemic. Some references to other similar work have been included.

*Line 70 - the four main findings need some additional detail as they lack clarity for example 'widespread support for alternative ethics review methods', how widespread, what alternative methods are being suggested - please add further details*

These four findings were verbatim quotes from the report so have now been formatted as such to make this clear. Indeed the whole purpose of the work reported here was to try to determine what the “alternative review methods” might be from the perspective of REC members.

*Line 77 - If public contributors are not always familiar or experienced with/in the practicalities of conducting research ethics reviews, or indeed research itself - then the question is why was this public dialogue undertaken? It seems there is a level of assumed knowledge needed from the reader about the HRA.*

This has been further clarified to state that multiple stakeholder engagements have been conducted, one of which was the public involvement exercise referenced in the introduction, and another was the consultation with REC members which is being reported in this manuscript.

*Line 82 - ongoing projects seeking the opinion of REC members - such as? What's the rationale for focusing on this one in particular?*

This has been clarified as above – one project was seeking public opinion, this manuscript is focusing on the project seeking REC members opinions.

*Line 83 -“REC members’ development days” - what are these?*

*Line 84 - These events were new to the HRA and their committees in 2022 - It is unclear which events are being referred to here. I am aware that training days for REC members existed even before the HRA existed, so would have assumed that a provision for REC members to come together existed under the HRA, prior to the pandemic - is this not the case? Perhaps some more detail of how the RECs operate and training etc is needed for the reader.*

*Line 91 - are the list of topics under discussion of relevance to the results? If not, you might want to remove this****.***

The description of the REC members days has been expanded, although the final comment (regarding old Line 91) is slightly inconsistent with the earlier request to describe what these days entail – but hopefully this section now reads a bit better.

*5. Review Comments to the Author*

*Abstract*

*Line 39-37 It is unclear how ‘attending annual training events’ is relevant here. Data were collected at 3 events.*

This has been clarified.

*Introduction*

*Line 49 State ‘Research Ethics Committees’ in full before using (REC).*

*Line 56 It would be helpful to define ‘research waste’.*

Both now done.

*Lines 60+ It would be useful here to provide an overview of the HRA review process. E.g., who are applicants, types of studies that require REC approval (as opposed to HRA only), panel members, role of the chair, use of ERF system etc. This will be common knowledge for most UK researchers who recruit via the NHS but not for those who don’t, or international readership, whose processes are likely to vary.*

This description has been significantly expanded as requested.

*Material & Methods*

*Lines 100+ Most elements of a traditional methodology section are missing, namely headings and information related to: design, setting/participants, materials, procedure, analysis. Please reformat to ensure that all key elements of the methodology are present, clear, and replicable.*

Headings have been added, and care taken to ensure that the description is sufficient to allow another research team to replicate the activities described here.

*More information is required re. group facilitation and note taking. It is unclear why audio recording was not utilised so this should be considered as a limitation of the methodology.*

*The nature of the note taking needs for description. Exactly what was noted? Was this verbatim or summarised in the researchers’ words?*

The method used has been expanded and the consequent limitations described more fully. However, as noted in the introductory paragraph of this letter, this manuscript is reporting on an activity originally designed for service improvement and not research *per se*, and thus while we are confident that the results are robust, we acknowledge that they do not follow standard research practice.

*Line 125 A full description of the process of content analysis is required. I also note that this method of analysis is not supported by a reference. Which form of content analysis was used?*

*It is unclear how consensus themes were formed when analysis was conducted following two different procedures (NVivo/manual) – please provide more information. It is also unclear what manual means.*

Thank you for this comment. You are absolutely correct that some confusion crept in by us referring to “themes”, so we have now significantly tidied up the results and discussion section to make sure it is clear that we conducted a content analysis and thus inductively identified “categories” of comments, rather than classified comments into pre-defined themes. We have referenced both a classic paper on the complexities of this approach alongside a very recent paper in the journal Research Ethics that conducted a similar analysis to the one we have done here. Regarding “manual coding” this was more a passing (and perhaps overly honest) reflection of trouble that one author had operating NVivo, meaning that the other researcher had to manually load all the categories into the software. Reference to this has now been deleted as despite taking a long time, it did not have any meaningful impact on the results.

*Results*

*Table 1. Best practice would be to collapse representation categories by region only, not by individual REC. For some RECs you have just 1 representative. Whilst it is unlikely that individuals’ can be identified, knowing that 1 member from a particular panel participants allows for process of elimination.*

*It is also unclear how these data were obtained as the methodology does not provide an overview of this data capture.*

Both done – table categories collapsed, and a line added to the methods describing how this data was obtained.

*Line 134+ I note that much of the text here relates to respondents mentioning the procedures that underpin the ethical review process but not what your respondents perceived as facilitators/barriers to making ethical decisions. My assumption is that there is more context within the response data re. why the discussion is important, the importance of ‘consistency’, weighing up risk/benefit etc. I recommend revisiting the data and trying to provide a richer overview of the responses.*

I think this reflects the challenges of this type of data collection and analysis. A significant weakness of our method (now explicitly stated and discussed) was the use of note takers who often did not provide the full context of specific comments. We have therefore tried to draw out as much as this as we can, and added a few more direct quotes from the data, but are mindful of other feedback that you have given about not using our own experience to overly interpret the data. However, should the reader be interested in trying to glean further context, the data has now been included in supplementary materials.

*Line 136 Careful with use of terminology such as ‘theme’. We would expect this term to be referenced in a thematic analysis less so in a content analysis if this type.*

*Table 3. The theme names here provide little information without also containing a definition of the theme and/or a response example (this comment is relevant to all other theme tables.)*

Have replaced all use of the word “theme” with “category” to reflect that this is a content, not thematic, analysis.

*Line 157 ‘it was interesting’ – best practice is to avoid commentary/interpretation and state findings factually in the results section.*

Deleted in the results, although kept a couple instances of the word “interesting” in the discussion where we feel it is more acceptable to provide interpretation.

*Line 162 The MHRA process will be unknown to most researchers. I think this highlights why it is important to provide an overview of the REC remit in the introduction, including its joint role with other agencies (e.g. MHRA, HMPPS etc.)*

Done

*General comment:*

*As with my comment for Question 1, I imagine there is a lot more to unpack here other than respondents mentioning the same thing. What is it about these things that the respondents deemed important? E.g., ‘checking the clarity of the participant facing information was also a distinct theme’ – why? I also note that theme headings appear to overlap between Questions 1 and 2. I wonder if some further analysis is required here to move responses to the appropriate question? It is usual for respondents to provide comments to a question which are more relevant to a subsequent question.*

*It is accepted practice to re-group data where appropriate.*

*For example, I see ‘role of chair’ is a theme in Q2 but I imagine this response is more relevant to Q1.*

We have provided some comment on this, although as mentioned, because of the limitation of using note takers we have been quite strict with ourselves in trying not to over interpret the data. As a consequence we have kept the results and initial discussion focussed purely on the data that actually came in under each heading, although our overall conclusions does take a slightly broader view of ideas that were expressed across the five questions (see point below regarding the four “actions” that the HRA will be taking away from this work).

*Line 167-168 ‘a concern that committees need to stay focussed on fundamental issues, rather than being distracted by minor issues’ – this information is difficult to interpret if the reader does not understand the ethical review process (i.e. contentious ethical issues are discussed at panel and applicants receive comprehensive feedback on these and other minor issues in writing – this would be useful in the introduction).*

*Like 172 -174 This is presented as though it is a quotation, yet it is a researcher summary of responses, it should be clear that this is the case (this comment is relevant to all comment excerpts).*

*Lines 180-190 I see the ERF information is provided here which is great. I still recommend a simple description in the introduction, considering the ERF is mentioned in a previous question response.*

*201-202 What was/wasn’t seemed as useful about the headings?*

All four points clarified in the text.

*Discussion*

*General comment:*

*There is much more detail provided re. the findings in the discussion than in the results section, where we should expect to see much of this summary (see comments about detail above). It would be useful to present the more of the discussion within the context of the literature base. My assumption is that the authors are very experienced REC members and much of the interpretation is personal reflection. It might be helpful to at least refer to REC terms of reference or policy documents when referring to process (assuming these are publicly accessible).*

We have tried to make the discussion a bit more factual and added some more references. However, it should be noted that as a service improvement exercise, the outcome as far as the HRA are concerned will/has been succinctly summarised in their internal report (not published externally) as being:

1) Provide more training on how to have a constructive discussion within the committee, updating the Ethics Review Form (ERF) to help guide such discussion.

2) Review the structure of study documents to ensure the material given to committees is more consistent

3) Continue working towards greater diversity among committee members, and where this is not possible strengthen the requirement for “Public and Patient Involvement and Engagement”.

4) Provide more feedback to committees on their performance.

We have therefore structured the discussion around these four points, showing how the data has led to these recommendations. Indeed as these conclusions are generalisable well beyond the HRA RECs, one big motivation for submitting this manuscript was to highlight the evidence for these so that they could be used elsewhere. We are not aware that this particular journal allows summary boxes, but would be happy to include these four recommendations in a more obvious way if the reviewer feels they are not displayed suitably. They are, however, stated in the abstract.

*Line 329-333 Reference missing for these findings.*

Reference added.

*General comment:*

*Much of the discussion provided in the latter part of this section contains important information to aid HRA developments. However for a manuscript of this type I would expect to see application of these findings to policy for RECs in general. Are you able to apply these findings to other agencies E.g., university RECs, HMPPS, international agencies?*

See above comment – we do think these findings are broadly generalisable across other RECs (and IRBs), and hence the reason for this paper.

*Line 374+ Please consider other limitations, e.g., the nature of the data collection.*

Now added as described above.

*(final comment from editor):*

*While revising your submission, please upload your figure files to the Preflight Analysis and Conversion Engine (PACE) digital diagnostic tool, https://pacev2.apexcovantage.com/. PACE helps ensure that figures meet PLOS requirements. To use PACE, you must first register as a user. Registration is free. Then, login and navigate to the UPLOAD tab, where you will find detailed instructions on how to use the tool. If you encounter any issues or have any questions when using PACE, please email PLOS at figures@plos.org. Please note that Supporting Information files do not need this step.*

This has been done and the modified figure file from the *PACE* tool uploaded.

We once again thank the reviewers for their comments that we do feel have genuinely improved our manuscript, and hope that it is now in a position to be published.

Yours Sincerely,

Simon, Matt and Clive.
